# Advances in metabolome information retrieval: turning chemistry into biology. Part II: biological information recovery

**DOI:** 10.1007/s10545-017-0080-0

**Published:** 2017-08-25

**Authors:** Abdellah Tebani, Carlos Afonso, Soumeya Bekri

**Affiliations:** 1 Department of Metabolic Biochemistry, Rouen University Hospital, 76000 Rouen, France; 2 Normandie Université, UNIROUEN, CHU Rouen, IRIB, INSERM U1245, 76000 Rouen, France; 3 Normandie Université, UNIROUEN, INSA Rouen, CNRS, COBRA, 76000 Rouen, France

**Keywords:** Omics, Metabolomics, Metabolome, Mass spectrometry, Nuclear magnetic resonance, Chemometrics

## Abstract

This work reports the second part of a review intending to give the state of the art of major metabolic phenotyping strategies. It particularly deals with inherent advantages and limits regarding data analysis issues and biological information retrieval tools along with translational challenges. This Part starts with introducing the main data preprocessing strategies of the different metabolomics data. Then, it describes the main data analysis techniques including univariate and multivariate aspects. It also addresses the challenges related to metabolite annotation and characterization. Finally, functional analysis including pathway and network strategies are discussed. The last section of this review is devoted to practical considerations and current challenges and pathways to bring metabolomics into clinical environments.

## Introduction

**Fig. 1:**
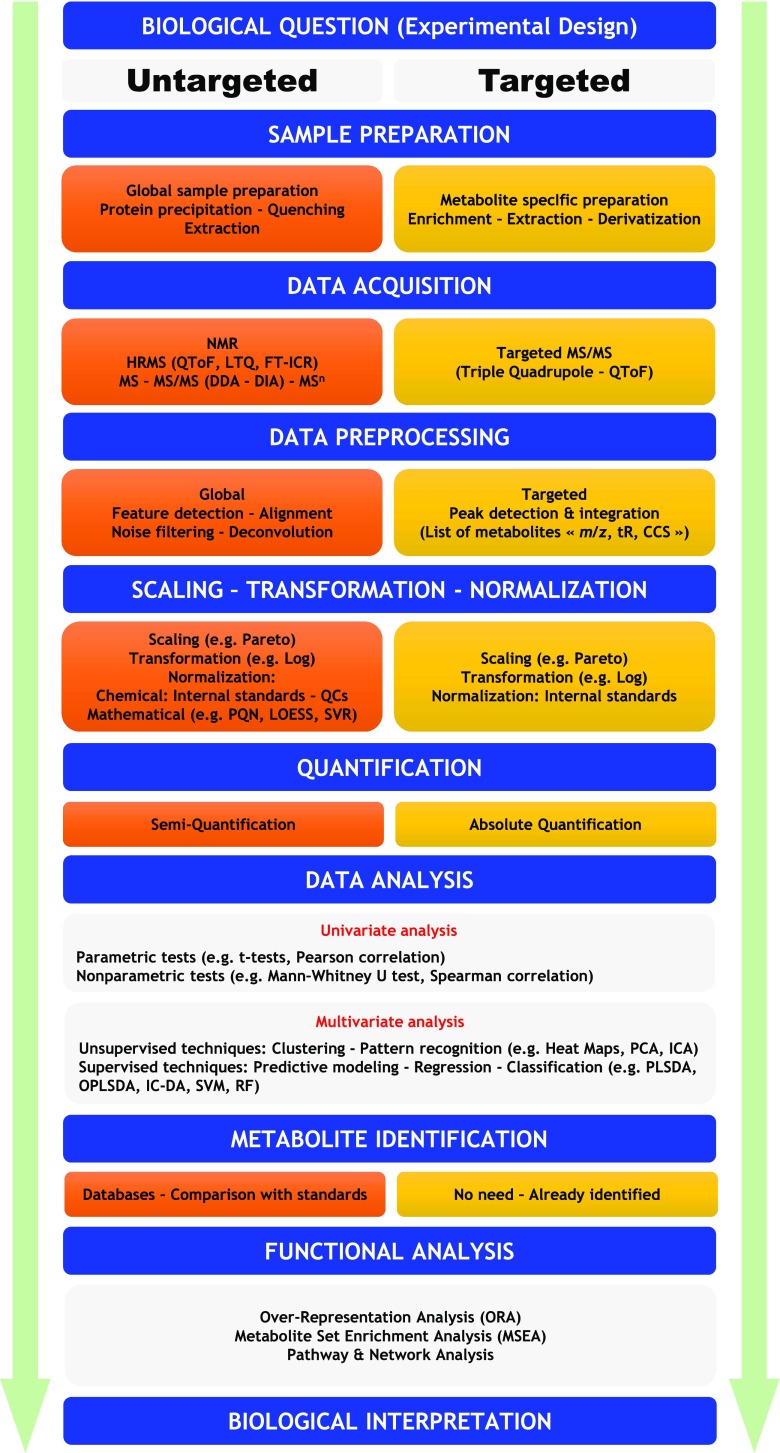
General metabolomics workflow. Metabolomics is divided into two main strategies. A targeted metabolomics is a quantitative analysis or a semiquantitative analysis of a set of metabolites that might be linked to common chemical classes or a selected metabolic pathway. An untargeted metabolomics approach is primarily based on the qualitative or semiquantitative analysis of the largest possible number of metabolites from diverse chemical and biological classes contained in a biological sample. The generated data undergo the data analysis step (univariate and multivariate) and functional analysis to get actionable biological insight

Addressing biology as an informational science is a key driver to translate biological data into actionable knowledge. This requires innovative tools that allow information extraction from high dimensional data. Bioinformatics is the field that was born to tackle this challenge (Hogeweg 2011). Bioinformatics applies informatics techniques such as applied mathematics, computer science, and statistics to retrieve the organized biological information. In short, bioinformatics is a management information system for a biological system (Luscombe et al 2001). The metabolomic data requires adapted statistical tools to retrieve as much chemical information as possible to translate it into biological knowledge. The major challenge is to reduce the dimensionality by selecting informative signals from the noise. To achieve this goal, chemometric tools are widely used. Chemometrics is the science of extracting useful information from chemical systems using data-driven means (Brereton 2014). It is inherently interdisciplinary, borrowing methods from data-analytic disciplines such as statistics, signal processing, and computer science. Descriptive and predictive problems could be addressed using chemical data. This second part of the review intends to give the state of the art of metabolomics data handling strategies along with their inherent advantages and limits regarding data analysis issues. Furthermore, biological information retrieval tools and their translational challenges into actionable results are described. Finally, practical considerations and current challenges to bring metabolomics into the clinical environment are discussed. The general metabolomics workflow is presented in Fig. 1.

## Biological information recovery

The analytical performance improvements associated with metabolomics platforms have led to the generation of complex and high-dimensional data sets. Handling the huge amount of generated data in a smoothly high-throughput fashion is a very important issue for transforming the data into clinically actionable knowledge.

### Preprocessing

Targeted metabolomics aims to process data sets retrieved from a subset of the metabolome. It contains predefined, chemically characterized and biochemically annotated metabolites. The main advantages of targeted metabolomics are that no analytical artifacts are carried throughout the downstream analysis; only a set of selected metabolites are analyzed. However, in untargeted metabolomics, data analysis is quite time-consuming. Different automated processes have been developed (Tsugawa et al 2013, 2014; Cai et al 2015) along with commercial solutions from instrument vendors. In contrast, the untargeted approach attempts a comprehensive analysis of all measurable metabolites in a given sample, including unknowns. It requires a holistic analysis of high-dimensional raw data sets, which in turn requires reducing the data into more computationally manageable formats without significantly compromising the contained chemical information. Because of noise, sample variation, or analytical/instrument factors, NMR and MS spectra often show differences in width, position, and peak shape. The goal of preprocessing is to correct these differences for better quantification of metabolites and enhanced intersample comparability. Data preprocessing includes some or all of the following steps: noise filtering, baseline correction, peak detection, peak alignment, and spectral deconvolution. Several preprocessing considerations and methods can be applied to both NMR and MS data (Vettukattil 2015; Szymanska et al 2016; Yi et al 2016). MS data preprocessing includes some or all of the following steps: noise filtering, baseline correction, peak detection, peak alignment, and spectral deconvolution. The order of the steps may differ between algorithms. Noise filtering is often applied to MS data to improve peak detection. Many different noise filters exist, including Gaussian, Savitzky–Golay, and wavelet-based filters (Yi et al 2016). The aim of the peak detection and deconvolution step is to identify and quantify the signals that correspond to the analytes (metabolites) in a given sample. Peak detection algorithms follow two strategies: derivative techniques or matched filter response (Szymanska et al 2016; Yi et al 2016). A deconvolution step is used to separate overlapping peaks in order to improve peak detection (Johnsen et al 2017). Furthermore, a de-isotoping step is used to cluster the isotopic peaks corresponding to the same chemical feature to clean the data matrix. Alignment of the detected features across different samples aims to remove intersample shifts, and several alignment algorithms have been developed (Smith et al 2013; Szymanska et al 2016). The data dimensionality has to be reduced to make them applicable to instruments paired with MS. Different strategies enable data compression such as binning and the “search of regions of interest (ROI)” methods that are the most adequate hyphenated MS data sets. A comparison of some peak-picking algorithms used in untargeted MS-based metabolomics have been reported (Rafiei and Sleno 2015). XCMS is an open access mass spectrometry data processing software. It is widely used in the metabolomics community. It was developed in response to the growing need for user-friendly software to process complex untargeted metabolomic data (Smith et al 2006; Gowda et al 2014). It has been designed as a solution for the entire untargeted metabolomic workflow ranging from the raw data processing to direct metabolite assignment through integrated and automated METLIN database queries. The platform has been recently upgraded with data streaming capabilities to support high-throughput, cloud-based data processing, and systems biology analyses (Huan et al 2017). NMR data preprocessing typically includes baseline correction, alignment, and binning. Baseline correction aims to correct systematic baseline distortion. Some spectral regions, such as that of water, are often removed. Peak shifts due to differences in instrumental factors such as salt concentrations, temperature, and pH changes can be corrected by alignment procedures (Smolinska et al 2012). Binning or bucketing is a dimension reduction method that splits the spectra into segments or bins and assigns a representative value to each bin. However, binning can hamper spectral resolution. The typical output of the preprocessing step is a data matrix that contains the detected features and the corresponding intensity (abundance) in each sample.

### Normalization

As with other omics, metabolomics data have several intrinsic characteristics, such as their asymmetric distribution (De Livera et al 2012) and a substantial proportion of instrumental, analytical, and biological noise (Grun et al 2014; Mak et al 2015). Thus, the goal of data normalization is to eliminate experimental biases related to the abundance of detected features between samples without compromising biological variations. Most of the methods are inspired by previous omic strategies (genomics and transcriptomics) that suffer from similar experimental biases (Tebani et al 2016). Indeed, the chemical diversity of metabolites and interindividual variations lead to changes in extraction and MS ionization yields, making it difficult to distinguish changes of biological interest from analytical biases (instrumentation, operators, and reagents). Strategies for normalization of metabolomics data can be divided into statistical approaches and chemical approaches. Statistical approaches are based on statistical models that define correction factors specific to each sample from the complete data set (Li et al 2016), such as normalization by standard deviation (Scholz et al 2004), mean global intensity (Wang et al 2003), quantile normalization (Lee et al 2012), probabilistic quotient normalization (Dieterle et al 2006), cyclic loess (Dudoit et al 2002), QC-robust spline batch correction (Kirwan et al 2013) or support vector regression (Shen et al 2016). Chemical approaches are based on one or more reference compounds (Hermansson et al 2005; Bijlsma et al 2006; Sysi-Aho et al 2007), internal standards, or endogenous or exogenous compounds that are used to normalize the entire chromatogram (single compound) or certain regions of the chromatogram by normalizing each zone according to a standard that is eluted in that region. Other strategies based on the characteristics of the studied matrix, such as dry mass of the samples, volume (e.g., 24-h urine), and osmolality. Protein or creatinine levels can also be used (Wu and Li 2016). A comprehensive comparison of state-of-the-art normalization techniques was recently reported (Li et al 2016).

### Transformation, centering, and scaling

Statistical methods assume that the data under analysis have a specific type of probability distribution. Thus, the inferences made from the data depend on the chosen distribution. If the data under examination do not exhibit that distribution, then the inferences could be false or misleading. Most parametric methods in metabolomics assume that the data have a Gaussian distribution. However, in metabolomics, MS and NMR data are hampered by noise from different sources. Furthermore, the feature distributions can be skewed. So, transformations aim to correct for heteroscedasticity and skewness before statistical analysis. This allows building of statistically meaningful and interpretable models in metabolomics. Different mathematical transformations can be used, such as log transformation and power transformation (van den Berg et al 2006). Multivariate analytical methods are based on latent variable projections that extract information from the data by projecting observations onto the direction of the maximum variance. Hence, NMR and MS data analysis by these methods mainly focuses on the average spectrum. This approach may mask underlying biological variation because more abundant metabolites will exhibit high values in the data matrix and subsequently show large differences among samples compared to less abundant metabolites. Data scaling methods divide each data point for a given feature by a scaling factor that is a measure of data dispersion for that feature. Therefore, scaling the data aims to remove the offset from the data and focus on the biological variation regarding similarities and dissimilarities of samples. There are several scaling methods such as auto-scaling (unit variance scaling), in which the mean and the standard deviation of the feature are calculated. The aim of auto-scaling is to give equal weights to all features, but this method is very sensitive to large deviations from the sample mean. Thus, pareto scaling is the most popular alternative in metabolomics. In pareto-scaling, each observation in the mean-centered feature is divided by the square root of the standard deviation. Pareto scaling is a compromise between mean-centering and auto-scaling (van den Berg et al 2006).

## Data analysis

### Univariate data analysis

Univariate statistical methods can be used in metabolomics. Their main limitation is that they consider only one variable at a time, which may not be convenient for high-dimensional data. Parametric tests such as Student’s *t*-test and ANOVA are commonly applied to assess the differences between two or more groups, respectively, provided that the normality assumption is verified (Broadhurst and Kell 2006). Otherwise, if normality is not assumed, a nonparametric test such as Mann–Whitney *U* test or Kruskal–Wallis one-way ANOVA can be used. Another important issue is that applying multiple univariate tests in parallel with a high-dimensional data set raises the multiple testing problem. Since a large number of features are simultaneously analyzed in metabolomics, the probability of accidentally finding a statistically significant difference (i.e., true positive) is high. Different correction methods can be used to handle this multiple testing issue. In the Bonferroni correction, the significance level for a hypothesis is divided by the number of hypotheses simultaneously being tested (Broadhurst and Kell 2006). Hence, the Bonferroni correction is considered a conservative correction method. Less conservative methods are available and are based on lowering the false-discovery rate (FDR). Less restrictive approaches FDR-based methods minimize the expected proportion of false positives among the total number of positives (Benjamini and Hochberg 1995). It should be noted that potential confounding factors such as sex, age, or diet may lead to spurious results if not properly addressed. Furthermore, the main disadvantage of univariate methods is their lack of feature correlations and insights about interactions. Hence, advanced multivariate approaches are more suitable for in-depth inferences.

### Multivariate data analysis

Bioinformatics a field that permits data collection, analysis, parsing, and contextual interpretation, and it supports decision-making on those bases. Bioinformatics can be defined as conceptualizing biology in terms of molecular components and by applying “informatics techniques” borrowed from disciplines such as applied mathematics, computer science, and statistics to understand and organize information on a large scale (Luscombe et al 2001). The major challenge is to reduce the dimensionality by selecting informative metabolic signals from the highly noisy raw data. Chemometric tools are widely used to achieve this goal. Chemometrics is defined as the science of extracting useful information from chemical systems by data-driven means (Brereton 2014). It may be applied to solve both descriptive and predictive problems, using biochemical data. In multivariate methods, representative samples are presented as points in the space of the initial variables. The samples can then be projected into a lower dimensionality space based on components or latent variables, such as a line, a plane, or a hyperplane, which can be seen as the shadow of the initial data set viewed from its best perspective. The sample coordinates of the newly defined latent variables are the scores, while the directions of variance to which they are projected are the loadings. The loadings vector for each latent variable contains the weights of each of the initial variables (metabolites) for that latent variable. Unsupervised methods attempt to reveal patterns or clustering trends in the data that underpin relationships between the samples. These methods also highlight the variables that are responsible for these relationships, using visualization means. Chemometrics methods are mainly divided into unsupervised and supervised methods. In unsupervised methods, no assumptions are made about the samples and the aim is mainly exploratory. In metabolomics data, metabolic similarity shapes the observed clustering. Principal component analysis (Hotelling 1933) is a widely used pattern recognition method; it is a projection-based method that reduces the dimensionality of the data by creating components. Principal component analysis allows a two- or three-dimensional visualization of the data. Because it contains no assumptions on the data, it is used as an initial visualization and exploratory tool to detect trends, groups, and outliers. It allows simpler global visualization by representing the variance in a small number of uncorrelated latent variables. Independent component analysis (ICA) is another unsupervised method that is a blind source separation method that separates multivariate signals into additive subcomponents (Bouveresse and Rutledge 2016). Its interpretation is similar to PCA, but instead of orthogonal components, it calculates non-Gaussian and mutually independent components (Wang et al 2008; Al-Saegh 2015). Compared to PCA, ICA as a linear method could provide potential benefits for untargeted metabolomics. ICA has been successfully used in metabolomics (Li et al 2012; Monakhova et al 2015; Liu et al 2016). Other unsupervised methods, such as clustering, aim to identify naturally occurring clusters in the data set by using similarity measures defined by distance and linkage metrics (Wiwie et al 2015). A dendrogram or a heat map can be created to visualize the similarities between samples. Commonly used clustering methods include correlation matrix, k-means clustering (Hartigan and Wong 1979), hierarchical cluster analysis (Johnson 1967), and self-organizing maps (Kohonen 1990; Goodwin et al 2014). In supervised methods, samples are assigned to classes or each sample is associated with a specific outcome value, and the aim is mainly explanatory and predictive. When the variables are discrete (e.g., control group versus diseased group), the task is called classification. When the variables are continuous (e.g., metabolite concentration) the task is called regression. The main purposes of supervised techniques are (i) to determine the association between the response variable and the predictors (metabolites) and (ii) to make accurate predictions based on the predictors. In metabolomics biomarker discovery, within the modeling process, it is important to find the simplest combination of metabolites that can produce a suitably effective predictive outcome. The biomarker discovery process involves two parameters, the biomarker utility and the number of metabolites used in the predictive model. The main challenges are therefore predictor selection and the evaluation of the fitness and predictive power of the built model. Predictor selection aims to identify important metabolites from among the detected ones that best explain and predict the biological or clinical outcome. Different predictor selection techniques have been described. Some of these suggested strategies are based on univariate or multivariate statistical proprieties of variables used as filters (loading weights, variable importance on projection scores, or regression coefficients), while others are based on optimization algorithms (Saeys et al 2007; Yi et al 2016). Recently, another elegant method has been reported that essentially combines estimation of Mahalanobis distances with principal component analysis and variable selection using a penalty metric instead of dimension reduction (Engel et al 2017). This method was successfully applied for inherited metabolic diseases (IMD) screening purposes. Finally, we need goodness-of-fit metrics to assess the model predictive power. Commonly used statistics may include root mean square error (RMSE) for regression problems and sensitivity, specificity, and the area under the receiver-operating characteristic (ROC) curve for classification models. To have independent test data sets, sometimes, data collection may be expensive or hampered by limited samples such as in rare diseases which is the case in IMD. In this case, various resampling methods are used to efficiently use the available data set, such as cross-validation, bootstrapping, and jackknifing (Westad and Marini 2015). Regarding the supervised methods, various techniques can be used in metabolomics. Some of the most used techniques include linear discriminant analysis (LDA) (Balog et al 2013; Ouyang et al 2014) and partial least squares (PLS) methods such as PLS-discriminant analysis (PLS-DA) (Wold et al 2001) and orthogonal-PLS-DA (OPLS-DA) (Trygg and Wold 2002; Manwaring et al 2013), as well as support vector machines (Cortes and Vapnik 1995; Lin et al 2011) and random forest (Breiman 2001; Huang et al 2015). Recently, Habchi et al proposed an innovative supervised method based on ICA called IC-DA. This method has been successfully applied to analyze DIMS metabolomics data that could be useful for high throughput screening (Habchi et al 2017). Furthermore, new methods based on topology data analysis are drawing interest and seem promising for data analysis because of their intrinsic flexibility and exploratory and predictive abilities (Liu et al 2015; Offroy and Duponchel 2016). Recently, a new method, called statistical health monitoring (SHM), has been adapted from industrial statistical process control; an individual metabolic profile is compared to a healthy one in a multivariate fashion. Abnormal metabolite patterns are thus detected, and more intelligible interpretation is enabled (Engel et al 2014). This approach has been successfully applied in IMD investigations (Engel et al 2017). The aim of metabolomics studies and the data analysis strategy are highly interdependent. Moreover, multivariate and univariate data analysis pipelines are not mutually exclusive, and they are often used together to enhance the quality of the information recovery. For further details on data analysis techniques and tools used in metabolomics, the reader may refer to recent reviews on this issue (Gromski et al 2015; Ren et al 2015; Misra and van der Hooft 2016).

## Metabolite annotation and characterization

The identification of the discriminant metabolites is an important step in metabolomics. The introduction of high-resolution mass spectrometers and accurate mass measurements that facilitate access to the chemical formula of the detected peaks has considerably accelerated this step. The combined use of quadrupole ion traps for sequential fragmentation experiments provides additional structural information needed to identify metabolites of interest. However, MS using soft ionization techniques such as electrospray methods, exhibits high variability in the fragmentation profiles generated on different devices due to the lack of standardized ionization conditions, thus limiting the construction of universal spectral data banks such as those obtained by electron ionization or NMR (Cui et al 2008). This issue could be addressed using standardized ionization conditions such as electron based ionization techniques that are highly reproducible across MS systems worldwide and across different vendors. Indeed, in MS, one or more chemical formulas can be generated if high-resolution instruments are used, which provides a first element for carrying out an interrogation of the existing databases. The acquisition of fragmentation spectra at this stage enables us to discriminate the responses obtained previously on the basis of the produced ions or neutral losses, characteristic of chemical groups. Given the importance of the identification step, standardization elements have been proposed to harmonize metabolite identification data. Thus, identification standards have been defined within the framework of the Metabolomics Standards Initiative according to the available information on the metabolite to be characterized (Sumner et al 2007). Computational tools such as CAMERA (Kuhl et al 2012), ProbMetab (Silva et al 2014), AStream (Alonso et al 2011), and MetAssign (Daly et al 2014) have been developed for metabolite annotation. These methods mainly use *m/z*, retention time, adduct patterns, isotope patterns, and correlation methods for metabolite annotation. However, in MS the detected *m/z* ion and MS database matching is insufficient for unambiguous charcterization. Although retention time prediction are still used to improve identification confidence, complementary orthogonal information is required for reliable assignment of chemical identity, such as retention time matching and molecular dissociation patterns compared to authentic standards (Sumner et al 2007). For reliable characterization, a solution may be in a multidimensionnal framework based on orthogonal information integration, which may include accurate mass *m/z*, chromatographic retention time, MS/MS spectra patterns, CCS, chiral form, and peak intensity. Furthermore, hybrid strategies, including pathway network and analysis methods, could enhance metabolite characterization through different metrics integration, including data-driven network topology, chemical features correlation, omics data, and biological databases. Such a multidimensional approach may permit the chemical characterization by merging both extended chemical information and biological context. The Human Metabolome Database (HMDB) was first introduced in 2007 and is currently the most comprehensive, organism-specific metabolomic database. It contains NMR and MS spectra, quantitative, analytical, and physiological information about human metabolites. It also contains associated enzymes or transporters and disease-related properties. The HMDB is a fully searchable database with many built-in tools for viewing, sorting and extracting metabolites information features. In addition, the HMDB also supports the direct identification of potential diagnostic biomarkers based on their accurate mass, mass spectra or NMR spectra. Hence, the HMDB is a valuable support for translational metabolomics to support biomarker discovery. Perhaps, the HMDB (Wishart et al 2013) is one of the most valuable databases for IMD investigations. Other databases are presented in Table 1.

**Table 1:** Biological databases and functional analysis tools

Tools	Websites	References
Biological databases		
KEGG (Kyoto Encyclopedia of Genes and Genomes)	http://www.genome.jp/kegg	(Kanehisa et al 2016)
HumanCyc (Encylopedia of Human Metabolic Pathways)	http://humancyc.org	(Romero et al 2005)
MetaCyc (Encyclopedia of Metabolic Pathways)	http://metacyc.org	(Caspi et al 2008)
Reactome (A Curated Knowledgebase of Pathways)	http://www.reactome.org	(Vastrik et al 2007)
SMPDB (Small Molecule Pathway Database)	http://www.smpdb.ca	(Jewison et al 2014)
Virtual Metabolic Human Database	https://vmh.uni.lu	(Thiele et al 2013)
Wikipathways	http://www.wikipathways.org	(Kelder et al 2012)
Pathway and networks analysis and visualization		
BioCyc—Omics Viewer	http://biocyc.org	(Caspi et al 2016)
iPath	http://pathways.embl.de	(Yamada et al 2011)
MetScape	http://metscape.ncibi.org	(Karnovsky et al 2012)
Paintomics	http://www.paintomics.org	(Garcia-Alcalde et al 2011)
Pathos	http://motif.gla.ac.uk/Pathos	(Leader et al 2011)
Pathvisio	http://www.pathvisio.org	(Kutmon et al 2015)
VANTED	http://vanted.ipk-gatersleben.de	(Rohn et al 2012)
IMPaLA	http://impala.molgen.mpg.de	(Kamburov et al 2011)
MBROLE 2.0	http://csbg.cnb.csic.es/mbrole2	(Lopez-Ibanez et al 2016)
MPEA	http://ekhidna.biocenter.helsinki.fi/poxo/mpea	(Kankainen et al 2011)
Mummichog	http://clinicalmetabolomics.org/init/default/software	(Li et al 2013)
PIUMet	http://fraenkel-nsf.csbi.mit.edu/PIUMet/	(Pirhaji et al 2016)
3Omics	http://3omics.cmdm.tw/	(Kuo et al 2013)
InCroMAP	http://www.ra.cs.uni-tuebingen.de/software/InCroMAP/	(Wrzodek et al 2013)
Multifunctional tools		
MetaboAnlayst	http://www.metaboanalyst.com	(Xia et al 2015)
XCMS online	https://xcmsonline.scripps.edu	(Tautenhahn et al 2012)
MASSyPup	http://www.bioprocess.org/massypup	(Winkler 2015)
Workflow4Metabolomics	http://workflow4metabolomics.org	(Giacomoni et al 2015)
Metabox	https://github.com/kwanjeeraw/metabox	(Wanichthanarak et al 2017)

## Functional analysis: translating information into knowledge

**Fig. 2:**
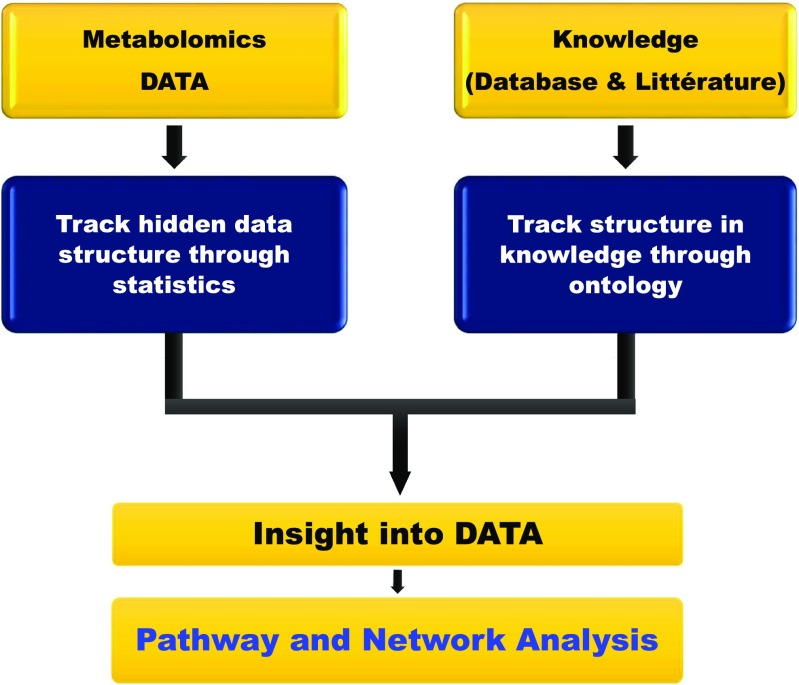
An illustration of pathway analysis strategies. Metabolome pathway analysis is designed to uncover significant pathway–phenotype relationships within a large data set. On one hand, it unveils hidden data structure in experimental data through differential expression using statistical metrics. On the other hand, it uses prior knowledge retrieved through biological databases and literature. Pathway analysis combines these two pillars to interpret the experimental findings

One of the fundamental difficulties in pathophysiological studies is that diseases might be caused by various genetic and environmental factors and their combinations. In addition, if a disease is caused by a combinatorial effect of many factors, the individual effects of each component might be low and thus hard to unveil. So, considering systems approaches to get deeper and informative biological insights is appealing. Any biological network can be pictured as a collection of linked nodes. The nodes may be genes, proteins, metabolites, diseases, or even individuals. The links or edges represent the interactions between the nodes: metabolic reactions, protein–protein interactions, gene–protein interactions, or interactions between individuals. The distribution of nodes ranges from random to highly clustered. However, biological networks are not random. They are collections of nodes and links that evolve as clusters; therefore, biological networks are referred to as scale-free, which means that they contain few highly-connected nodes called hubs. The core idea of the biological network theory is the modularity structure. Three distinct modules can be defined: topological, functional, and disease modules (Barabasi et al 2011). A topological module represents a local subset of nodes and links in the network; in this module, nodes have a higher tendency to link to nodes within the same local neighborhood. A functional module is a collection of nodes with similar or correlated function in the same network zone. Finally, a disease module represents a group of network components that together contribute to a cellular function whose disruption results in a disease phenotype. Of note, these three modules are correlated and overlap. Computational biology is gaining increasingly more space in modern biology to embrace this new network perspective. It can be divided into two main fields: knowledge discovery (or data-mining) and simulation-based analysis. The former generates hypotheses by extracting hidden patterns from high-dimensional experimental data. However, the latter tests hypotheses with in silico experiments, yielding predictions to be confirmed by in vitro and in vivo studies (Kitano 2002). Thus, pathway and network analysis strategies rely on the information generated by metabolomics studies for biological inference (Thiele et al 2013; Cazzaniga et al 2014). Both approaches exploit the interrelationships contained in the metabolomic data. Network modeling and pathway-mapping tools help to decipher the roles of metabolite interactions in a biological disturbance (Cazzaniga et al 2014). Biological databases are important for mapping different metabolic pathways (Table 1). Conceptual framework of pathway analysis is illustrated in Fig. 2. Indeed, pathway analysis or metabolite set enrichment analysis (MSEA) are methodologically based on the gene set enrichment analysis approach, previously developed for pathway analysis of gene-expression data (Khatri et al 2012; Garcia-Campos et al 2015). There are three distinct methods for performing MSEA: overrepresentation analysis (ORA), quantitative enrichment analysis (QEA), and single-sample profiling (SSP) (Xia and Wishart 2010; Garcia-Campos et al 2015; Xia et al 2015). An important advantage of computational metabolomics lies in the use of correlations among feature signals to map chemical identity. Since metabolites are interconnected by a series of biochemical reactions to build the network of metabolites, they can be interrogated using network-based analytical tools. In metabolomics, network analysis uses the high degree of correlation in metabolomics data to build metabolic networks based on the complex relationships of the measured metabolites. Based on the observed relationship patterns in the experimental data, correlation-based methods allow building metabolic networks in which each metabolite represents a node. However, unlike the pathway analysis, the links between nodes denote the level of mathematical correlation between each metabolite pair and are called edge (Krumsiek et al 2011; Valcarcel et al 2011; Do et al 2015). These data-driven strategies have been successfully applied for the reconstruction of metabolic networks from metabolomics data (Krumsiek et al 2011; Shin et al 2014; Bartel et al 2015). Biological inference often needs prior identification of metabolites. Since this step is challenging, a novel approach, named Mummichog, has been proposed by Li et al to reboot the conventional metabolomic workflow (Li et al 2013). This method predicts biological activity directly from MS-based untargeted metabolomics data without a priori identification of metabolites. The idea behind this strategy is combining network analysis and metabolite prediction under the same computational framework, which significantly reduces the metabolomics workflow time. Based on spectral peaks, the computational prediction of metabolites yields several hits; thus, a “null” distribution can be estimated by how these predicted metabolites, retrieved from a metabolomics experiment, map to all known metabolite reactions through interrogating databases. Despite most annotations being false, the biological meaning underpinning the data drives enrichment of the metabolites. The metabolite enrichment pattern of real metabolites compared to the null distribution is then statistically assessed. This method has been elegantly illustrated in an exploration of innate immune cell activation, which revealed that glutathione metabolism is modified by viral infection driven by constitutive nitric oxide synthases (Li et al 2013). Recently, Mummichog has been used for metabolic pathway analysis in a population by untargeted metabolomics. Hoffman et al identified metabolic pathways linked to age, sex, and genotype, including glycerophospholipid, neurotransmitters, metabolism carnitine shuttle, and amino acid metabolism (Hoffman et al 2016). Tyrosine metabolism was found to be associated with nonalcoholic fatty liver (Jin et al 2016). Pirhaji et al described a new network-based approach using a prize-winning Steiner forest algorithm for integrative analysis of untargeted metabolomics (PIUMet). This method infers molecular pathways via integrative analysis of metabolites without prior identification. Furthermore, PIUMet enabled elucidating putative identities of altered metabolites and inferring experimentally undetected, disease-associated metabolites and dysregulated proteins (Pirhaji et al 2016). Compared to Mummichog, PIUMet also allows system-level inference by integrating other omics data. Contextualization of metabolomics information is also important in pathophysiological investigations. From a metabolic network stand point, flux is defined as the rate (i.e., quantity per unit time) at which metabolites are converted or transported between different compartments (Aon and Cortassa 2015). Thus, metabolic fluxes, or the fluxome, represent a unique and functional readout of the phenotype (Cascante and Marin 2008; Aon and Cortassa 2015). Thus, from a network view of metabolism, one or more metabolic fluxes could be altered in a given metabolic disorder depending on the complexity of the disease (Lanpher et al 2006). To interrogate these fluxes, fluxome network modeling can be achieved using constraints of mass and charge conservation along with stoichiometric and thermodynamic limitations (Cortassa and Aon 2012; Winter and Kromer 2013; Kell and Goodacre 2014; Aurich and Thiele 2016). Based on the stoichiometry of the reactants and products of biochemical reactions, flux balance analysis can estimate metabolic fluxes without knowledge about the kinetics of the participating enzymes (Cascante and Marin 2008; Aon and Cortassa 2015). Recently, Cortassa et al suggested a new approach, distinct from flux balance analysis or metabolic flux analysis, that takes into account kinetic mechanisms and regulatory interactions (Cortassa et al 2015).

Since metabolites are often involved in multiple pathways, biologically-mediated labeling is particularly informative in such cases. Given the dynamics and compartmentation that characterize the metabolism, isotopic labeling is poised as an appealing approach to unambiguously track metabolic events. Advances in atom-tracking technologies and related informatics are disruptive for metabolomics-based investigations thanks to their contextual high throughput information retrieval. Among these technologies, stable isotope resolved metabolomics (SIRM) is a method that allows tracking individual atoms through compartmentalized metabolic networks which allowed highly resolved investigations of disease-related metabolomes (Higashi et al 2014; Fan et al 2016; Kim et al 2016). A wide variety of software tools are available for analyzing metabolomic data at the pathway and network levels. Table 1 presents different functional analysis tools for both pathway analysis and visualization.

## Metabolomics and other omics cross-talk

**Fig. 3:**
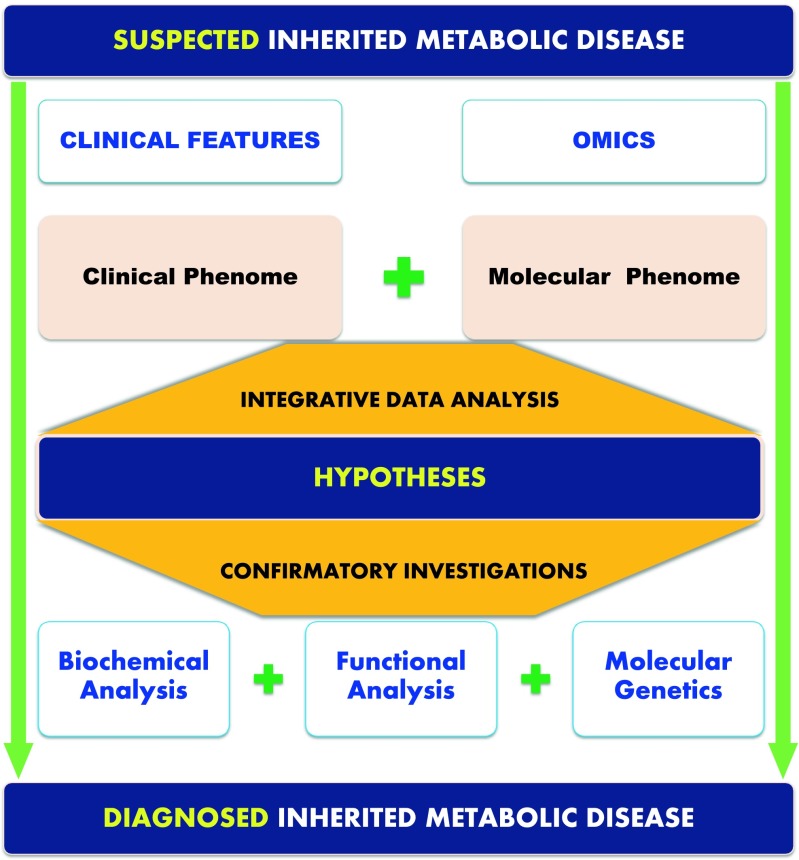
Paradigm shift in inherited metabolic diseases investigation. High-throughput analytical technologies, integrative bioinformatics, and medical computational frameworks will allow intelligible molecular and clinical information recovery and effective medical decision-making

Since IMD are associated with a genetic defect, their current characterization addresses both the mutated gene and its products. Currently, understanding of genetic variation effects on phenotypes is limited in most IMD which leads to consider the influence of genetic or environmental modifying factors and the impact of an altered pathway on metabolic flux as a whole. These diseases are related to the disruption of specific interactions in a highly organized metabolic network (Sahoo et al 2012; Argmann et al 2016). Thus, the impact of a given disruption is not easily predictable (Lanpher et al 2006; Cho et al 2012). Therefore, functional overview, integrating both space and time dimensions, is needed to assess the actors of the altered pathway and the potential interactions of each actor (Aon 2014). Thus, metabolomics combined with genome-wide association studies (mGWAS) track genetic influences on metabotypes which underpin the human’s metabolic individuality (Suhre et al 2016). Unveiling the genetically influenced metabolic variations could raise huge potential pathophysiological studies (Shin et al 2014). This includes functional understanding of clinical outcomes and genetic variation associations, designing targeted therapies for metabolic disorders and also identification of genetic modifiers underlying metabolic disease biomarkers. Different studies have reported genetic influences of metabotypes, disease-risk biomarkers or drug response variations (Suhre et al 2016). In a recent study, Rhee et al analyzed the association between exome variants and 217 plasma metabolites in 2076 participants in the Framingham Heart Study, with replication in 1528 individuals of the Atherosclerosis Risk in Communities Study. They identified an association between guanosine monophosphate synthase and xanthosine using single variant analysis and associations between histidine amonia lyase (HAL) and histidine, phenylalanine hydoxylase (PAH) and phenylalanine, and ureidopropionase (UPB1) and ureidopropionate using gene-based tests, which highlights novel coding variants that may unveil inborn errors of metabolism (Rhee et al 2016). Shin et al reported a comprehensive study exploring genetic loci influences on human metabotypes in 7824 individuals from two European cohorts, KORA (Germany) and Twins (UK), using MS-based metabolomics. They mapped significant associations at 145 loci and their metabotype connectivity through more than 400 blood metabolites. The built model unveiled information on heritability, gene expression and overlap with known complex disorders and inborn errors of metabolism loci. The data were used to build an online database for data mining and visualization (Shin et al 2014). The effectiveness of multi-omic approaches has been recently illustrated by van Karnebeek et al. The authors reported a disruption of the N-acetylneuraminic acid pathway in patients with severe developmental delay and skeletal dysplasia using both genomics and metabolomics approaches. Variations in the *NANS* gene encoding the synthase for N-acetylneuraminic acid were identified (van Karnebeek et al 2016). This elegantly highlights how systemic approaches may address IMD complexity and allow their diagnosis (Argmann et al 2016). For more details on mGWAS studies, the reader may refer to recent reviews (Kastenmuller et al 2015; Suhre et al 2016). Figure 3 shows how laboratory workflow using high-throughput analytical technologies, integrative bioinformatics, and computational frameworks will reshape IMD investigations. This integrative approach will allow intelligible molecular and clinical information recovery for a more effective medical decision-making in IMD.

## Perspectives in clinical metabolomics translation

Despite spectral information becoming available in the literature or in spectral databases, metabolite identification is still a challenging task (Goodacre et al 2007). However, metabolite identification remains a central issue in metabolomics prior to embracing complete clinical translation. No software is currently available to automate the identification step. Furthermore, metabolite identification is mandatory for absolute quantitation especially in MS-based methods requiring the use of stable isotope-labeled internal standards. Some data-driven alternatives have been developed to elucidate metabolite structure associations such as correlation-based network and modularity analysis. The association structure can be used to identify MS ions derived from the same metabolite (Broeckling et al 2014) or to identify biotransformations (Kind and Fiehn 2010). However, these knowledge-based approaches may be hampered by their limits for addressing the entire chemical space and limited coverage of metabolome databases. Another limitation lies in the cost for targeted analyses, which cannot reasonably be expected to support measurement of tens of thousands of chemicals in large populations. Thus, more efforts are needed to overcome this issue. However, in IMD a few hundred key metabolites may be defined for large-scale screening. Standardized and validated protocols are a prerequisite for metabolic phenotyping technologies. Harmonization of the sample preparation, processing, analysis, and reporting, using validated and standardized protocols, is mandatory (Chitayat and Rudan 2016; Kohler et al 2016). Standardized protocols are particularly helpful for untargeted metabolomics. In targeted methods, since each analyte is known and quantified, technology versatility is less important. Despite substantial efforts to standardize untargeted metabolomics methods, there are still no universally adopted protocols, particularly for MS-based strategies. This situation is due to the diverse and ever-changing analytical platform. The community and journals may take a lead in standardization by aligning it to community-published standards, such as the Metabolomics Standards Initiative (Sumner et al 2007), and data repisotories to encourage open metabolomic data, such as MetaboLights database at the EBI. All these endeavors aim to develop infrastructures and frameworks standardize terminology, data structure, and analytical workflows (Levin et al 2016). Finally, addressing these standardization issues is essential for regulatory compliance, which is a prerequisite for any clinical implementation. Automation at different stages, at instrument and pre- and post-analytic levels, is an important issue for broader use of metabolomics technologies. Automation enhances throughput, reproducibility, and reliability. Direct infusion MS-based methods are currently used for newborn screening in routine clinical practice (Therrell et al 2015; Ombrone et al 2016). Moreover, they are also taking the lead from a translational perspective, such as the iKnife, which would allow real-time cancer diagnosis (Balog et al 2013), and breathomics strategies for lung and respiratory diseases based on breath signatures (Hauschild et al 2015). Furthermore, metabolomics generates a huge amount of data that require comprehensive analysis and integration with other omics and metadata to infer the topology and dynamics of the underlying biological networks. Advanced statistical and computational tools along with effective data visualization are required to smoothly handle the diversity and quantity of the data and metabolite mapping (Alyass et al 2015; Ritchie et al 2015). In this regard, combining genomic and metabolic information may enhance biological inference and even clinical diagnostics (Tarailo-Graovac et al 2016; van Karnebeek et al 2016). Despite these promising steps, further advances in computational tools are needed for more efficient storage and integration (Perez-Riverol et al 2017).

## Conclusion

Translating metabolomic data into actionable knowledge is the ultimate goal. Particular attention should be paid to computational tools for multidimensional data processing. There is an urgent need for more databases with validated and curated MRM transitions for targeted metabolites. Furthermore, for untargeted metabolomics, larger libraries and curated MS/MS spectra for metabolite identification are needed. Hybrid strategies including pathway and network analysis methods could enhance metabolite characterization through integration of different metrics, including data-driven network topology, chemical features correlation, omics data, and biological databases. Such multidimensional approaches may improve the chemical characterization by combining both extended chemical information and biological context. With all the high-dimensional data management issues, like other omics, metabolomics clinical implementation should be tackled using big data handling strategies for efficient storage, integration, visualization, and sharing of metabolomics data. To achieve the promise of the Precision Medicine era, it is crucial to combine expertise from multiple disciplines, including clinicians, medical laboratory professionals, data scientists, computational biologists, and biostatisticians. This raises the urgent need to think about new teams with new skill sets and overlapping expertise for more effective medical interactions across all healthcare partners for the management of IMD. Training the next generation medical workforce to manage and interpret omics data is a way to go and inception of such thinking has already started (Henricks et al 2016).
